# Phyllosphere of senescent crops as a microbial N_2_O source

**DOI:** 10.3389/fmicb.2025.1650612

**Published:** 2026-01-26

**Authors:** Kanako Tago, Shin-Ichi Tokuda, Yuya Sato, Yasuyo Sekiyama, Yong Guo, Yong Wang, Yasuhiro Date, Shintaro Hara, Megumi Kuroiwa, Tsubasa Ohbayashi, Luciano Nobuhiro Aoyagi, Tomoyasu Nishizawa, Shigeto Sudo, Yuichi Suwa, Masahito Hayatsu

**Affiliations:** 1 Institute for Agro-Environmental Sciences, National Agriculture and Food Research Organization (NARO), Tsukuba, Japan; 2 School of Veterinary Medicine, Kitasato University, Sagamihara, Japan; 3 Central Region Agricultural Research Center, NARO, Tsukuba, Japan; 4 Environmental Management Research Institute, National Institute of Advanced Industrial Science and Technology (AIST), Tsukuba, Japan; 5 Research Center for Advanced Analysis, NARO, Tsukuba, Japan; 6 College of Agriculture, Ibaraki University, Ami, Japan; 7 Institute for Plant Protection, NARO, Tsukuba, Japan; 8 TanBIO Inc., Tsukuba, Japan; 9 Department of Biological Sciences, Chuo University, Bunkyo-ku, Japan; 10 Graduate School of Engineering, Tokyo University of Agriculture and Technology, Koganei, Japan

**Keywords:** crop residue, denitrification, electron donor, microbes on phyllosphere, nitrous oxide emission, senescent leaf

## Abstract

Plant residues contribute to the nitrogen cycle in terrestrial ecosystems, as they are recognized as a nutrient source for soil microorganisms. However, the contribution of the microbial community in the phyllosphere of plant residues, such as senescent leaves, itself in the nitrogen cycle remains unclear. In agricultural lands, crop residues contribute to global emissions of the greenhouse gas nitrous oxide (N_2_O), which is an intermediate product of several microbial pathways including nitrification and denitrification. We examined direct N_2_O emissions from aboveground cabbage leaf residues via denitrification by indigenous microbial communities inhabiting the phyllosphere of the residue. We conducted a variety of experiments, ranging from field experiments to multi-omics analyses. We found that cabbage leaves accumulated nitrate from 3.0 to 11.3 NO_3_^–^-N mg g^–1^ leaf dry weight by application of chemical fertilizers and/or cow manure compost. Leaf senescence triggered N_2_O emissions (8.62–415.35 μg N_2_O–N m^–2^ h^–1^), and denitrifiers from five genera were isolated from the senescent leaves. A representative denitrifier, *Agrobacterium* sp. 6Ca8 utilized leaf nitrate as an electron acceptor and carbon sources such as glucose, succinate, and pyroglutamate as electron donors to produce N_2_O. Strain 6Ca8 co-expressed genes for denitrification and aerobic respiration, as well as genes for central metabolic pathways, providing key precursors essential for ATP production and cellular biosynthesis. Our findings elucidate the role of the residual plant phyllosphere as a microbial hotspot of N_2_O emissions in agricultural fields. This is the study demonstrating denitrifiying bacteria present on leaves and their functions as drivers of N_2_O production. Furthermore, we demonstrate that denitrification, which is known as an anaerobic process, can occur by utilizing nutrients released from senescent leaves, even on the leaf surface surrounded by air. Our study expands the ecological niche of denitrification from belowground soil to aboveground plants in terrestrial ecosystems.

## 1 Introduction

The nitrogen cycle is important for regulating primary productivity and species diversity in terrestrial ecosystems. However, this is becoming unbalanced owing to excessive amount of nitrogen produced via anthropogenic nitrogen fixation (Canfield et al., 2010). Extensive nitrogen input via synthetic fertilizers and manure applications (Tian et al., 2020) in agricultural fields results in increased emissions of the greenhouse gas nitrous oxide (N_2_O) (Tian et al., 2019), which is responsible for stratospheric ozone depletion (Ravishankara et al., 2009).

Crop residues, or senescent crops, provide another source of N_2_O emissions. In fields where significant amounts of crop residues remain after harvest or where organic residues are added, the decomposition of senescent crops plays a pivotal role in the nitrogen cycle (Haynes, 1986). The amount of major crop residues exceeds that of agricultural production and is estimated to be approximately 3.7 Pg dry matter year^–1^ globally (Bentsen et al., 2014). Global N_2_O emissions in relation to crop residues were estimated at approximately 224 Mt CO_2_-eq year^–1^ in 2017, contributing 9% of the total, global agricultural N_2_O emissions.^1^ N_2_O emissions from crop residues vary substantially with the crop and soil type (Novoa and Tejeda, 2006; Akiyama et al., 2020). For example, 3.02–7.5% of the nitrogen input via unharvested cabbage and potato residues are emitted directly from the soil as N_2_O-N in Andosol and Fluvisol fields (Akiyama et al., 2020).

Denitrification is a microbial anaerobic respiratory process in which available carbon and inorganic nitrogen (i.e., nitrate or nitrite) are used as electron donors and acceptors, respectively, to produce gaseous nitrogen (i.e., nitric oxide, N_2_O, and N_2_). Senescent crops release nitrogen and water-soluble decomposable carbon, which can be used as energy sources for denitrifiers in the soil. Thus, most relevant studies have treated N_2_O emissions from crop residues as soil-based events (Chen et al., 2013; Cui et al., 2021). However, the leaf surfaces of corn, soybean, and switchgrass decomposing in soil serve as local hotspots for denitrification (Kravchenko et al., 2017; Kim et al., 2021). Studies on N_2_O mitigation in soybean fields have revealed that senescent nodules, rather than soil inorganic nitrogen, are the main nitrogen source for N_2_O flux, indicating that senescent crops can serve as substrates for nearby denitrifiers (Itakura et al., 2012; Akiyama et al., 2016). In a no-till corn field, only 0.08 g of a decaying pigweed leaf accounted for 85% of the total denitrification activity in an intact 98-g soil core (Parkin, 1987). These results suggest that direct N_2_O emissions via denitrification in crop residues occur independently of soil denitrification.

Emissions of N_2_O from the phyllosphere of cropping systems have been demonstrated (Zou et al., 2005; Rochester et al., 2015). Plants appear to transport N_2_O derived from the soil, or produce it in their tissues either through nitrate assimilation during photosynthesis or via endophytic bacteria and other abiotic or unknown mechanisms (Baggs and Philippot, 2011; Lenhart et al., 2019). However, denitrification has received less attention; there is an example of denitrifiers isolated from sphagnum moss (Nie et al., 2015). In aquatic environments, nutrient release via decomposition of submerged macrophytes contributes to denitrification by epiphytic microbes (Han et al., 2019). Crops accumulate carbon and nitrogen substrates due to nitrogen fertilization. We hypothesized that if denitrifiers could access crop nutrients, N_2_O emissions would occur in the phyllosphere of senescent crops.

Research on plant-associated microbes in terrestrial ecosystems has been expanding from the belowground rhizosphere, including traditional symbiotic relationships via plant roots (e.g., mycorrhizal fungi and rhizobia) and pathogenic interactions, to the phyllosphere, including plant health, nutrient cycling and ecological adaptation to climate change. Advances in high-throughput sequencing technologies have help unveil the composition and function of microbes in the phyllosphere (Zhu et al., 2021). Recent studies have shown that a significant number of microbes remain on the phyllosphere of dead plants in forests and streams (Leach et al., 2017; Hayer et al., 2022), which has a significant impact on litter decomposition rates (Fanin et al., 2021). If phyllosphere microbes on fresh leaves persist throughout leaf senescence, they may have a significant advantage over exogenous microbes in the initial colonization of the decomposing leaf (Austin et al., 2014). Plant quality is a major factor influencing nitrogen dynamics in soil through decomposition, which also influences N_2_O emissions from crop residues (Chen et al., 2013; Berg and McClaugherty, 2014). In general, nitrogen release occurs when the C/N ratio of the litter is lower than 20 (Brady and Weil, 2013), and N_2_O emissions significantly increase when the C/N ratio of the crop residue is lower than 25 (Charles et al., 2017). Thus, if indigenous microbes on crop leaf have the capability to denitrification, N_2_O emissions can occur simultaneously with crop senescence. However, microbial functions during senescence are far less understood. Moreover, the nitrogen cycle has seldom been linked to the decomposition of senescent leaves by their associated phyllosphere microbes.

The capability for denitrification, reduction of nitrate to nitrogen gas, is widespread among bacteria, fungi, and archaea, and the underlying molecular mechanisms via four reductases—Nar or Nap, Nir, Nor, and Nos—are well characterized (Zumft, 1997). High-throughput genetics has demonstrated the modularity of denitrification pathways (Graf et al., 2014) and revealed novel N_2_O reduction genes (Jones et al., 2014; Shan et al., 2021). Meanwhile, although available carbon is the primary substrate driving denitrification, along with nitrate and nitrite, no such substrates have yet been identified in the field. Moreover, the ecological significance of plant-derived components as carbon sources for the electron supply remains unclear. To understand the exact mechanisms of denitrification in the field, carbon metabolism and nitrogen transformation must be evaluated simultaneously.

Our previous studies have shown N_2_O emissions during harvest season in soybean, potato, and cabbage crop fields (Itakura et al., 2012; Akiyama et al., 2020). Akiyama et al. (2020) showed that the annual emission factors (EFs) for cabbage and potato residues (3.02–7.51%, depending on soil types) were much higher than those from synthetic fertilizers (0.62%). Other reports have also shown that crop residues accounted for 73% of cumulative N_2_O emissions in cabbage fields (Koga et al., 2004) and 65% in lettuce fields (Baggs et al., 2000). These results indicate that residues from these crops substantially contribute to N_2_O emissions. In this study, we propose that the phyllosphere of plant residues is a hotspot of the nitrogen cycle independent of the soil, by focusing on N_2_O dynamics and the microorganisms involved in denitrification on the phyllosphere. We aimed to 1) assess the direct contribution of the phyllosphere of senescent leaves to N_2_O emissions, 2) identify the denitrifiers involved, and 3) describe the mechanisms underpinning these processes. We conducted field and laboratory (culture-based) experiments, to understand microbial processes in the field (Prosser, 2015). We combined transcriptomic and metabolomic analyses to determine the functional pathway responsible for N_2_O production via denitrification. Our study provides a comprehensive understanding of phyllosphere-based denitrification,—an overlooked but potentially important process responsible for N_2_O emissions and the microbial nitrogen cycle.

## 2 Materials and methods

### 2.1 Field experiment

An experimental cabbage (*B. oleracea* var. *capitata*) field (Supplementary Figure 1) was established in 2002 at the National Institute of Vegetable and Tea Science, NARO (36° 01’ N, 140° 06’ E; 21 m above sea level), with 18 plots (25 m^2^) of six treatments (Supplementary Figure 1A), arranged in a randomized complete block design (*n* = 3): NF, no fertilizer; PF, conventional fertilization combining chemical fertilizers and cow manure compost; CF, chemical fertilizers alone; MC-1, cow manure compost at 250 kg N ha^–1^; MC-2, cow manure compost at 500 kg N ha^–1^; and MC-3, cow manure compost at 750 kg N ha^–1^. The soil chemical characteristics and fertilizer N used for each treatment are shown in Supplementary Table 1.

Cabbage was cultivated in winter and summer in 2009 and 2010. N_2_O flux in the field experiment was measured using the static closed chamber method (Yan et al., 2001). The chamber was set on the ground to enclose a growing cabbage plant to measure N_2_O flux from the plant–soil system comprising both the soil and crop surfaces. Precipitation, as well as soil temperature, water content, pH, ammonia, and nitrate were measured (Supplementary Figure 2). To measure direct N_2_O flux from an unharvested leaf (Supplementary Figure 1B) *in situ*, unharvested leaves in the field were quickly transferred to a 17-L plastic container and closed. The amount of N_2_O gas accumulated in the container was measured.

### 2.2 Microcosm experiment

Unharvested outer cabbage leaves and soil were collected from the PF-treated plots. To imitate harvest-season field conditions, a microcosm was prepared (*n* = 6): two unharvested leaves were placed on 5 kg of soil in a 17 L plastic container. One leaf was placed on the other so that the upper one avoided adhesion to soil particles. The microcosms were incubated at 25°C. N_2_O flux from the microcosms was periodically measured during incubation. When the microcosms showed the highest N_2_O emission, the upper leaf was taken and cut into several pieces, and the N_2_O flux of the subsamples was measured to identify the most active point on the leaf surface, that is, the N_2_O-emitting hot spot.

### 2.3 N_2_O, oxygen, and chemical properties

N_2_O flux was measured using a Shimadzu GC-2014 gas chromatograph equipped with a thermal conductivity detector, or a Shimadzu GC-14B equipped with an electron capture detector (Shimadzu Co., Kyoto, Japan). Dissolved N_2_O and oxygen concentrations in liquid cultures were measured using microsensors for N_2_O and O_2_, respectively (Unisense, Aarhus, Denmark). The end product of denitrification was measured using the acetylene block method, and gas chromatography–mass spectrometry analysis using ^15^NO_3_^–^. Nitrate and ammonium concentrations were analyzed using the Cu–Cd reduction method and indophenol-blue method, respectively. Total organic carbon and nitrogen were measured using a TOC–V/TN analyzer (Shimadzu Co., Kyoto, Japan).

### 2.4 Culture conditions

Two media were used to screen for denitrifiers: R2A medium (Difco, BD, Franklin Lakes, NJ, United States) with 1 g L^–1^ potassium nitrate added (R2A-N) and a customized medium comprising cabbage extract (CE). To prepare the CE medium, cabbage leaves taken from the field were boiled in four volumes of water for 30 s to inactivate cellular enzymes and then homogenized with a blender. Plant debris in the crude extract was then removed by filtration through cotton gauze and filter paper, and the filtrate was autoclaved and stored at 4°C before use. The culture (2.5 mL) was incubated under static conditions in a 20 mL glass serum vial (Nichiden-Rika Glass, Kobe, Japan) at 25°C in the dark (*n* = 3). During the incubation, the cultures were not sealed, allowing air to pass naturally through the gas phase within the vial.

### 2.5 Screening and identification of denitrifiers

Bacterial cells were extracted from the N_2_O-emitting hotspots using a Nycodenz density gradient method (Ikeda et al., 2009). The cell suspension was serially diluted with CE or R2A-N liquid media, and denitrification activity was measured by determining N_2_O production. Denitrifiers were screened using the dilution-plate method. The isolated denitrifiers were classified via 16S rRNA gene sequencing analysis, using the bacterial universal primer set 27f and 1492r, as described elsewhere (Tago et al., 2015). N_2_O reductase activity of bacterial culture was measured via the acetylene block method (Tiedje, 1994).

### 2.6 Genome analysis

Total DNA was extracted from isolated denitrifiers belonging to *Agrobacterium* spp., using the Genomic-Tip 100/G kit (Qiagen, Valencia, CA, United States). Genome sequencing was performed using PacBio RSII single-molecule real-time (SMRT) sequencing technology (PacBio, Menlo Park, CA, United States) and an Illumina MiSeq platform (Illumina, Inc., San Diego, CA, United States). Read processing and hybrid assembly were performed as previously reported (Guo et al., 2020). Briefly, single-end reads were trimmed from the MiSeq paired-end reads using sickle v.1.33,^2^ with default settings. The Unicycler pipeline (v.0.4.8) (Wick et al., 2017) was used for combined assembly of the PacBio and the MiSeq reads, after which genome polishing was performed using the built-in tool Pilon (v.1.24) (Walker et al., 2014). Automatic annotation and identification of rRNA and tRNA genes and CDSs were performed using the DDBJ Fast Annotation and Submission Tool (DFAST) pipeline (Tanizawa et al., 2018), based on Prokka software (Seemann, 2014). The CDSs identified as “hypothetical proteins” by DFAST were manually identified and annotated against the NCBI non-redundant database using the BLAST.

### 2.7 RNA-seq analysis

A representative denitrifier, *Agrobacterium* sp. strain 6Ca8, was cultured in CE medium at 25°C under static conditions (denitrifying conditions) (*n* = 3). As a negative control, the culture was aerated by stirring at 60 rpm to restrict cellular denitrification activity. Under the aerated conditions (the negative control), only a small amount of dissolved N_2_O was detected in the culture (Supplementary Figure 3). Cells of 6Ca8 at the early- and mid-log phases (OD_600_ of approximately 0.4 and 0.9, respectively) were collected, and the total RNA was extracted and purified using an RNeasy kit (Qiagen, Valencia, CA, United States). The extracted nucleic acid was treated with TURBO DNase (TURBO DNA-free kit, Thermo Fisher Scientific, Waltham, MA, United States), followed by the Ribo-Zero rRNA Removal kit (Bacteria; Illumina, Inc., San Diego, CA, United States), according to the manufacturer’s protocols. The mRNA was purified using an RNA Clean & Concentrator-5 kit (Zymo Research, Irvine, CA, United States), according to the manufacturer’s protocols. mRNA quality was determined using a Bioanalyzer and a Prokaryote Total RNA Pico Chip (Agilent Technologies, Foster City, CA, United States).

For library preparation, the NEBNext Ultra RNA Library Prep Kit for Illumina and NEB Multiplex Oligos for Illumina (New England Biolabs, Ipswich, MA, United States) were used for fragmentation, adapter ligation, cDNA synthesis, and PCR amplification, according to the manufacturer’s protocols. The library was loaded on an agarose gel containing Synergel (Diversified Biotech Inc., Dedham, MA, United States; Synergel:agarose, 5:3), and the appropriate fragment size (200–300 bp) was purified using a gel extraction kit (Qiagen, Valencia, CA, United States). The final products were sequenced on an Illumina MiSeq platform (Illumina, Inc., San Diego, CA, United States). To ensure high sequence quality, the remaining sequencing adaptors and reads with a Phred quality score > 15 (or > 20, for leading and tailing sequences) and reads shorter than 80 bp were removed using Trimmomatic v.0.30, with Illumina TruSeq3 adapter sequences used for clipping (Bolger et al., 2014). The remaining paired reads were analyzed using FastQC^3^ for quality control, and Bowtie2 (v. 2.2.2) (Langmead and Salzberg, 2012) for mapping onto the 6Ca8 genome (DDBJ/EMBL/GenBank accession: AP026433–AP026435). After converting the output BAM files to BED files using the bamtobed function in BEDTools (v. 2.14.3) (Quinlan and Hall, 2010), gene expression levels were calculated as TPM using an in-house script (Sato et al., 2019). The data from early-log phase cells were further analyzed, because the difference in the expression of denitrification-related genes between the denitrifying and control (aerobic) conditions was larger for early-log than mid-log phase cells (Supplementary Table 2).

### 2.8 Identification of electron donors in crop residue

Cells cultured in CE medium (*n* = 3), at the initial and late-log phases, were filtered to remove cells and contaminants, then subjected to NMR analysis. The filtrate (140 μL) was added to 560 μL of 125 mM potassium phosphate buffer (pH or pD 7.2) in deuterium oxide (D_2_O, 99.9%, Cambridge Isotope Laboratories, Andover, MA, United States) containing 1.25 mM of 2,2-dimethyl-2-silapentane-5-sulfonate sodium salt (DSS, Sigma–Aldrich, St. Louis, MO, United States). NMR spectra were recorded on a Bruker AVANCE500 spectrometer (Bruker BioSpin GmbH, Rheinstetten, Germany) equipped with a dual carbon/proton CPDUL cryoprobe, that fits 5-mm-diameter NMR tubes, according to the previously described procedure (Li et al., 2021) with a slight modification. Data acquisition was done in acquisition mode with a spectral width of 20 ppm, in digital quadrature detection, with a proton 90° pulse value of 20 μs, offset frequency of 4.7 ppm, 4 s relaxation delay, 65,536 data points, and 128 scans. The metabolites were identified and quantified relatively using the Chenomx NMR Suite (Chenomx, Edmonton, Alberta, Canada).

The identified carbon sources consumed during cell growth in CE were further subjected to a culturing experiment to verify that they were electron donors for denitrification. Strain 6Ca8 was incubated in minimal medium with 5 mM carbon source and 10 mM potassium nitrate (*n* = 3). Cell growth and nitrate consumption for 20 h were measured. N_2_O production rate was measured using the cells at late-log phase. Concentrations of sugars and succinic acid were measured using the F-kit (sucrose/D-glucose/D-fructose and succinate; R-Biopharm, Darmstadt, Germany) according to the manufacturer’s protocol. Pyroglutamic acid content was determined using a Bruker AVANCE III HD 500 NMR spectrometer (Bruker BioSpin GmbH, Rheinstetten, Germany) equipped with a cryogenic probe. NMR measurements were performed using the Bruker standard pulse program “zgpr” with the above-mentioned acquisition parameters with a proton 90° pulse value of 15 μs. The obtained spectra were preprocessed and quantified on TopSpin software v.3.6.2 (Bruker BioSpin GmbH, Rheinstetten, Germany) based on the DSS internal standard.

### 2.9 Distribution of denitrifiers in senescent cabbage phyllosphere

The distribution and abundance of the isolated denitrifiers in senescent cabbage phyllosphere was characterized via amplicon sequencing and qPCR analyses. Frozen subsamples of fresh and senescent leaves were homogenized by bead beating using a Multi beads shocker MB-200 (Yasui Kikai, Osaka, Japan) at 2,000 rpm, and DNA was extracted using the Power Plant Pro DNA isolation kit (Qiagen, Valencia, CA, United States). To amplify the V4 region of the bacterial 16S rRNA gene, we modified the Earth Microbiome Project protocol to use 16S rRNA primers 515f (5’-GTGYCAGCMGCCGCGGTAA-3’) and 806r (5’-GGACTACNVGGGTWTCTAAT-3’), with the addition of a peptide nucleic acids (PNAs)-matching plastid (5’-GGCTCAACCCTGGACAG-3’) and mitochondrial DNA (5’-GGCAAGTGTTCTTCGGA-3’) (Lundberg et al., 2013). The qPCR analysis was completed using the StepOnePlus*™* Real-Time PCR system (ThermoFisher Scientific, Waltham, MA, United States).

For amplicon sequencing, the PCR products were purified using AMPure XP beads (Agencourt Bioscience, Beverley, MA, United States). A second PCR and sequencing was then carried out, according to the protocols supplied with the Illumina MiSeq platform. Using the QIIME 2 pipeline (v. 2021.4) (Caporaso et al., 2010) and the dada2 plugin (Callahan et al., 2017), the paired-end fastq files were processed via primer trimming, quality filtering, merging of paired ends, chimera removal, singleton removal, and construction of a feature table of ASVs. Because the quality scores of the sequences are lower at the ends of the reverse reads, the reverse reads were truncated to 263 bp using the “–p-trunc-len-r” option implemented in the dada2 plugin. Taxonomic identification of ASVs in the feature table was conducted using the SILVA database (release 138) (Quast et al., 2013), using the Qiime2 feature-classifier plug-in (Bokulich et al., 2018). ASVs classified as chloroplasts or mitochondria were removed. The sequence reads of each sample were rarefied to 5,684 reads per sample, and percentage relative abundance and alpha diversity were calculated using the QIIME2 pipeline.

### 2.10 Statistical analysis

Single-regression analyses were conducted using BellCurve for Excel (SSRI Inc., Tokyo, Japan) to analyze the relationships between N_2_O flux and field environmental factors. based on statistical analysis (significant level of 5%). The significance of the difference in the gene expression between the denitrification condition and control (aerobic condition) in RNA-seq analysis was tested with a *t*-test.

## 3 Results and discussion

### 3.1 Unharvested leaf as a microbial hotspot of N_2_O emission in cabbage fields

**FIGURE 1:**
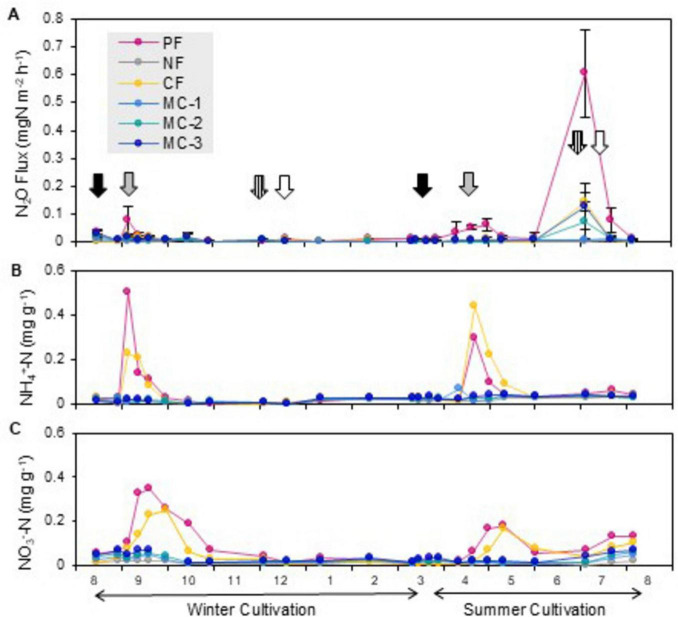
N_2_O flux and inorganic nitrogen under long-term treatment with manure, chemical fertilizer, or both in cabbage field. **(A)** N_2_O flux; **(B)** soil ammonium content; **(C)** soil nitrate content. Symbols: magenta, conventional fertilization with chemical fertilizer and cow manure compost (PF); gray, no fertilizer (NF); yellow, chemical fertilizer (CF); right blue, green, and dark blue, cow manure compost application at 250, 500, and 750 kg ha^– 1^, respectively, for total nitrogen (MC-1, -2, and -3). Black, gray, stripe and white arrows in **(A)** indicate manure application, chemical fertilizer application, harvest, and removal or incorporation of the unharvested residue, respectively.

We have shown that residues of soybean, potato, and cabbage crops contribute to N_2_O emissions during harvest season in these fields (Itakura et al., 2012; Akiyama et al., 2020). To understand whether the crop residue itself serves as a local hotspot of N_2_O emission, cabbage (*B. oleracea* var. *capitata*) was cultivated in winter and summer under six patterns of fertilizer application (Supplementary Table 1). During winter cultivation, the N_2_O flux occurred immediately after fertilization (0.077 mg N_2_O-N m^–2^ h^–1^) in the treatments with conventional fertilization (PF). N_2_O fluxes after fertilization was likely due to the accumulation of ammonium and nitrate in the soil (Figures 1B,C). During summer cultivation, the N_2_O flux reached a maximum of 0.064 mg N_2_O–N m^–2^ h^–1^ after fertilization in the treatments with PF. Another remarkably large flux occurred just after the harvest under summer cultivation in the treatments of PF (0.605 mg N_2_O–N m^–2^ h^–1^), chemical fertilization (CF) (0.143 mg N_2_O–N m^–2^ h^–1^), and cow manure compost fertilization with 500 kg N ha^–1^ (MC-2) (0.075 mg N_2_O–N m^–2^ h^–1^) and 750 kg N ha^–1^ (MC-3) (0.126 mg N_2_O–N m^–2^ h^–1^). This N_2_O peak could not be explained by soil ammonia and nitrate because their concentrations were low (Figures 1B,C). In contrast, nitrate accumulated in the unharvested outer leaves (Supplementary Table 1), and the leaf nitrate contents were highly positively correlated with cumulative N_2_O emissions during the summer cultivation harvest season (Table 1). N_2_O flux declined after cabbage residues were entirely removed from the field or when they were incorporated into the soil (Figure 1A, white arrow). These results suggest that leaf nitrate provides a direct link between unharvested aboveground residues and N_2_O emissions.

**TABLE 1:** Relationship between cumulative N_2_O emission and properties of soil and plant during harvest season under summer cultivation.

Variable	Correlation coefficient	*p*-value
N_2_O production activity	-0.196	0.7036
Total nitrogen content of soil	0.134	0.7958
Total carbon content of soil	0.170	0.7412
Volumetric water contents of soil	^–^0.086	0.8679
Ammonium content of soil	0.560	0.225
Nitrate content of soil	0.917	0.0036
pH (H_2_O) of soil	-0.577	0.2079
Dry weight of unharvested leaves	0.849	0.0184
Total nitrogen content of unharvested leaves	0.873	0.0117
Nitrate content of unharvested leaves	0.930	0.0023

To confirm direct N_2_O emissions from the phyllosphere of the residue, we measured N_2_O flux from the crop–soil system and unharvested leaves alone (Supplementary Figure 1B) in the PF treatment. Unharvested leaves produced N_2_O, with some leaves showing higher flux than the crop–soil system (Table 2), indicating that the unharvested leaves could be a source of N_2_O emissions.

**TABLE 2:** N_2_O flux from crop-soil system and unharvested leaf in three plots (A to C) of PF treatment.

Sample	Flux (μg N_2_O-N h^–1^ m^2–1^)
**Plot A**	
Crop-soil system*	200.19
Unharvested leaf	
Replicate 1	138.73
Replicate 2	188.53
Replicate 3	10.18
**Plot B**	
Crop-soil system*	109.96
Unharvested leaf	
Replicate 1	336.59
Replicate 2	8.62
Replicate 3	12.94
**Plot C**	
Crop-soil system*	45.38
Unharvested leaf	
Replicate 1	199.46
Replicate 2	162.19
Replicate 3	415.35

*A chamber was set on the ground to enclose a growing cabbage plant to measure N_2_O flux from the soil and crop surfaces.

To experimentally confirm the results of the field experiment, a microcosm was prepared with six replicates, using the soil and the unharvested leaves collected from the PF treatment. In five of the six replicates, N_2_O was emitted as the leaves became senescent and decomposed (Table 3), and the amount of emission varied depending on the position of the leaf. pH, which affects microbial activity, was higher in the senescent leaves (maximum pH 8.57) than in fresh leaves (average pH 5.47). Furthermore, bacterial populations, measured by qPCR of the bacterial 16S rRNA gene, were considerably larger in senescent leaves (> 10^8^ copies g^–1^ fresh wt) than in fresh leaves (at 10^5^ copies g^–1^ fresh wt). Nitrate remained in the senescent leaves. Nitrate was moderately and negatively correlated with N_2_O flux (correlation coefficient, *r* = –0.552; Supplementary Figure 4), suggesting that greater nitrate consumption was associated with higher N_2_O production. Although the results represented only a snapshot of the nitrate and N_2_O levels in senescent leaves, they suggest that nitrate may have been utilized for N_2_O production.

**TABLE 3:** N_2_O flux, and chemical and microbial characteristics of unharvest cabbage leaf.

Subsample ID*	Flux (μ g N*_2_*O-N h^–1^g^–1^ fresh wt)	pH	NO_3_^–^ (μ g g^–1^ fresh wt)		TOC (mg g^–1^ fresh wt)		TN (mg g^–1^ fresh wt)		16S rRNA gene copies (× 10^6^ copies g^–1^ fresh wt)		16S rRNA amplicon sequencing analysis					Photo of senescent leaf and subsample position
											Sequence reads		Number of ASVs^†^	Relative abundance of isolated denitrifiers in the sequence reads (%)		
			Mean	SD	Mean	SD	Mean	SD	Mean	SD	Total	Bacteria		6Ca8	5Ca50	
1_1	0.10	8.33	261.62	10.61	9.13	0.11	1.59	0.01	508.30	6.84	153,344	153,253	158	0.649	0.085	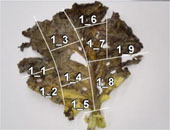
1_2	0.34	7.33	442.36	51.84	23.25	0.55	5.21	0.09	795.72	6.02	180,262	178,923	149	0.210	0.052	
1_3	1.23	8.38	226.09	19.24	11.53	0.20	1.74	0.04	153.01	0.47	167,116	165,735	120	1.524	1.837	
1_4	4.32	8.03	134.89	11.55	4.57	0.07	1.08	0.01	467.74	17.28	132,544	132,454	112	1.117	0.652	
1_5	1.04	7.2	202.87	2.05	6.44	0.13	1.78	0.01	1072.05	6.99	131,645	131,536	123	1.486	0.174	
1_6	0.88	8.04	129.44	13.71	4.65	0.07	0.88	0.01	649.03	7.12	139,107	139,042	169	1.713	0.268	
**1_7**	17.09	7.86	64.30	4.73	3.97	0.10	0.66	0.01	456.21	18.38	150,382	150,349	156	1.224	0.817	
1_8	9.31	7.85	64.06	4.44	4.09	0.06	0.85	0.01	2263.81	51.45	142,598	142,521	139	0.213	0.434	
1_9	1.06	7.83	491.86	26.29	11.42	0.19	2.86	0.04	1692.42	11.78	139,202	138,993	152	0.119	0.037	
2_1	4.73	7.7	160.71	9.40	6.70	0.09	1.55	0.01	222.25	6.58	164,026	163,165	168	0.444	0.767	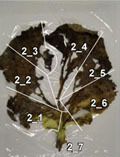
2_2	13.23	7.66	296.20	5.38	8.87	0.03	1.96	0.03	243.40	4.70	160,602	159,710	167	0.570	1.004	
2_3	0.78	8.33	264.93	19.30	11.60	0.11	2.17	0.03	594.10	6.73	138,170	137,406	164	0.191	0.080	
2_4	2.24	8.47	80.64	2.28	6.45	0.01	1.32	0.02	337.97	1.98	129,259	129,193	165	0.834	1.634	
2_5	2.37	7.93	340.74	15.20	8.84	0.05	2.43	0.04	1003.72	5.81	154,419	154,229	140	0.502	0.093	
2_6	6.73	7.62	256.64	19.34	10.27	0.13	2.60	0.03	410.89	3.46	151,106	150,575	120	0.104	0.036	
2_7	4.00	6.73	310.18	21.44	9.51	0.13	2.97	0.03	946.82	14.48	136,365	136,233	145	5.298	0.791	
3_1	16.90	8.19	53.64	3.36	4.62	0.02	1.08	0.02	929.79	9.85	136,760	136,714	146	0.191	0.230	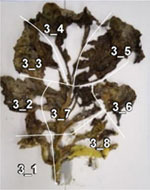
3_2	1.16	7.78	428.85	54.74	7.76	0.08	2.26	0.01	746.19	14.92	137,769	137,724	172	0.240	0.179	
3_3	0.22	8.46	280.81	25.13	8.76	0.08	1.81	0.01	608.87	2.30	146,414	146,148	182	0.510	0.133	
3_4	1.86	8.57	190.79	30.67	6.50	0.04	1.24	0.02	324.50	4.67	124,384	124,345	182	0.396	0.181	
3_5	2.09	8.02	146.26	10.86	5.54	0.05	1.15	0.01	424.31	11.38	128,430	128,380	193	0.426	0.079	
3_6	1.23	7.85	138.92	9.54	7.04	0.13	1.88	0.02	886.18	5.60	132,677	132,624	170	0.277	0.067	
3_7	15.53	8.17	28.29	3.36	3.76	0.04	0.88	0.03	469.74	6.17	133,721	133,695	180	0.756	0.123	
3_8	13.36	8.04	43.45	2.84	5.91	0.01	1.25	0.01	651.39	11.40	130,666	130,512	189	0.266	1.239	
5_1	0.08	7.82	185.02	11.89	4.46	0.04	1.03	0.01	189.35	4.18	149,787	149,756	147	1.387	0.751	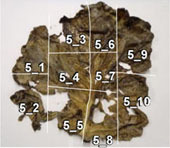
5_2	0.30	7.82	291.85	22.00	4.77	0.09	1.41	0.01	378.11	1.20	163,857	163,761	165	0.976	0.196	
5_3	1.98	8.01	177.56	15.08	3.34	0.03	0.91	0.01	577.87	6.27	157,311	157,308	177	0.831	0.341	
5_4	1.48	7.87	208.12	10.78	2.82	0.06	0.84	0.01	367.50	3.36	138,932	138,923	165	0.804	1.212	
5_5	0.89	7.76	480.67	31.94	4.83	0.05	1.70	0.03	409.12	2.95	133,366	133,361	163	1.669	3.362	
5_6	1.02	8.21	217.56	17.10	4.43	0.05	1.07	0.01	579.30	13.04	157,155	157,143	146	0.927	0.504	
**5_7**	6.43	8.21	133.59	13.53	2.80	0.04	0.85	0.01	208.21	11.81	152,509	152,478	189	1.260	0.433	
5_8	3.84	7.78	457.07	17.16	4.85	0.07	1.55	0.02	191.32	4.73	152,955	152,940	164	3.042	1.352	
5_9	0.69	8.19	326.63	12.68	5.85	0.08	1.44	0.01	176.22	5.09	142,570	142,478	172	1.743	1.396	
5_10	0.15	7.81	590.49	78.88	8.08	0.09	2.55	0.03	363.32	9.65	153,865	153,786	177	1.531	0.091	
6_1	7.97	6.72	333.59	20.59	13.59	0.11	3.41	0.03	1014.73	53.88	123,101	123,026	159	0.319	0.335	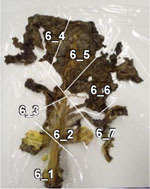
6_2	8.69	7.9	139.30	8.31	5.37	0.04	1.49	0.01	376.93	13.81	139,472	139,421	120	0.232	0.054	
**6_3**	11.89	8.41	9.61	0.86	3.49	0.05	0.60	0.02	461.32	8.00	146,048	146,040	153	0.242	3.172	
6_4	2.54	7.92	238.43	9.02	8.60	0.08	1.95	0.01	416.93	24.46	125,568	125,517	192	0.847	0.365	
6_5	5.13	8.03	262.04	34.98	7.87	0.04	1.85	0.01	549.56	2.84	119,666	119,607	181	0.727	3.373	
6_6	9.49	7.86	132.35	1.97	5.47	0.11	1.13	0.01	355.10	5.31	133,492	133,476	178	1.126	0.826	
6_7	4.20	7.57	238.18	16.45	7.94	0.09	1.71	0.03	1095.60	40.81	133,376	133,374	124	0.694	0.636	
Fresh 1^‡^	–	5.60	36.69	2.62	9.89	0.19	0.70	0.02	0.26	0.01	121,978	5,684	81	0.000	nd	
Fresh 2	–	5.28	207.87	9.70	4.01	0.06	0.55	0.01	0.62	0.03	147,839	16,535	87	0.000	nd	
Fresh 3	–	5.54	117.19	4.11	4.93	0.08	0.59	0.01	0.71	0.04	138,226	16,861	83	0.641	nd	

*The subsamples showing high N_2_O flux (1_7, 5_7, and 6_3, shown in bold), were used to isolate denitrifirers.

† Number of ASVs was rarefied to 5684 reads per sample.

‡ Characteristics of fresh cabbage leaves as controls.

Heading crops and leafy vegetables tend to accumulate nitrate in their body because of nitrogen fertilizer applications (Turan and Sevimli, 2005; Czech et al., 2012). Correlations between nitrate concentration and N_2_O emissions have been reported for some crop residues (Muhammad et al., 2011). After harvest, plant growth ceases and senescence proceeds; during the senescence, plant tissues disorganize, and degradation of chloroplast, protein, nucleic, and lipid is activated (Havé et al., 2017). Our results suggest that unharvested cabbage leaves contain nitrate, and that the leaf senescence could trigger the direct N_2_O emissions from its phyllosphere. During the senescence, nutrient release from the plant tissue, and microbes colonizing the leaf surface could then quickly access these nutrients in order to increase their populations. Under these circumstances, the senescent leaves could become a hotspot for N_2_O emissions, independently of soil.

N_2_O is produced via two microbial processes: nitrification and denitrification. Amplicon sequencing of bacterial community structure in the phyllosphere of senescent leaves revealed no detectable sequence reads corresponding to nitrifying bacteria (Supplementary Figure 5). As nitrate is the primary electron acceptor for denitrification, we postulated that N_2_O is directly emitted from the phyllosphere of senescent cabbage leaf, and denitrification is the main process responsible for its production. This suggestion can be elucidated by isolation and identification of denitrifiers functioning in senescent leaves and analysis of the metabolism of the leaf contents by the isolates.

### 3.2 Denitrifiers in the phyllosphere of senescent cabbage leaves

Although denitrification capability is widespread among plant-associated bacteria, it remains uncertain whether denitrification occurs in the phyllosphere. Our results suggest that the phyllosphere of senescent leaves provides favorable conditions for N_2_O production via denitrification. We therefore isolated denitrifiers from the senescent cabbage leaves showing high N_2_O flux, using two liquid media: a conventional R2A medium supplied with nitrate (R2A-N medium) and our unique CE medium made from cabbage leaf extract. The denitrifiers were classified into five genera (Supplementary Table 3): *Achromobacter*, *Agrobacterium*, *Alcaligenes*, *Brucella*, and *Stenotrophomonas*. Most of the denitrifiers isolated from the CE medium were classified into *Agrobacterium* spp., and they were further used to clarify the process of denitrification in senescent leaves. As *Agrobacterium* is one of the most common genera in the phyllosphere (Vorholt, 2012), it provides a suitable model for understanding the processes of denitrification and N_2_O emissions in senescent leaves.

**FIGURE 2:**
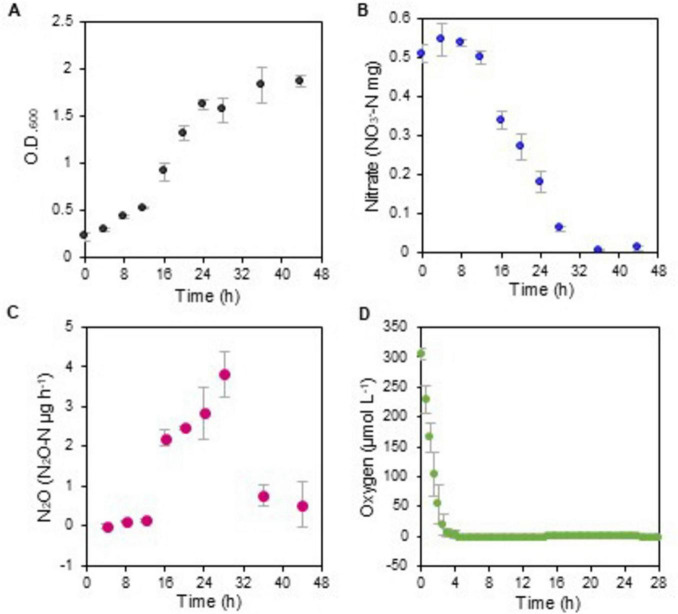
Growth of the denitrifying *Agrobacterium* sp. strain 6Ca8 in CE medium under static conditions. **(A)** Cell growth; **(B)** nitrate concentration; **(C)** N_2_O production per hour; **(D)** oxygen concentration in the culture. In **(C)**, the culture was sealed only when collecting N_2_O, as the culture was incubated under aerobic static conditions.

To evaluate N_2_O emissions by denitrifiers in the phyllosphere of senescent leaves, we cultured the representative isolate, *Agrobacterium* sp. strain 6Ca8, in CE medium under static conditions imitating the leaf environment. During cell growth, nitrate was consumed concurrently with the release of N_2_O (Figures 2A–C), indicating that this strain produces N_2_O using the nitrate from the cabbage extracts as an electron acceptor. Dissolved oxygen in the CE medium was consumed within 4.5 h of incubation (Figure 2D), indicating that the cells grew under conditions that were initially aerobic and subsequently anaerobic. This finding has also been reported for plant-associated denitrifiers such as *Agrobacterium fabrum* C58 (Baek and Shapleigh, 2005) and *Ensifer meliloti* (Torres et al., 2014). A transition from aerobic to anaerobic conditions may therefore be required for these plant-associated microbes to activate denitrification. Since the phyllosphere is subjected to stressful conditions, such as desiccation and rainfall, which alter water and oxygen contents, plant-associated microbes might need to change their respiration mode depending on the prevailing oxygen level.

### 3.3 Intracellular gene expression of aerobic respiration, denitrification, and central metabolic pathways

To reveal the intracellular molecular mechanisms of denitrification by senescent phyllosphere-derived denitrifiers, we sequenced the complete genome of *Agrobacterium* sp. strain 6Ca8 (Supplementary Figure 6A), followed by RNA-seq analysis. This revealed a series of denitrifying genes, *nap*, *nir*, and *nor*, encoding nitrate, nitrite, and nitric oxide reductases, respectively, in its genome (Supplementary Figure 6B). RNA transcripts of 6Ca8 cells in CE medium at the early-log phase (at 8 h; Figure 2A) were subjected to RNA-seq analysis. Expression of the nitrate reductase genes *napEFDABC* was 2.6–4.4-fold higher under denitrifying conditions than under aerobic conditions (Table 4). The expression of the nitrite reductase genes *nirVK* and nitric oxide reductase genes *norDQBCFE* increased more than 25.36-fold under denitrifying conditions, which were among the top 20 transcripts with the highest fold-change in expression from aerobic to denitrifying conditions (Supplementary Table 4). The expression of other genes related to electron biogenesis (coding cytochromes, quinols, and pseudoazurin) and of those related to Nir and Nor regulation (*fnrN*, *sinR*, *nnrR*, *nnrS*, and *nnrU*) (Zumft, 1997; Baek et al., 2008) increased by up to 60-fold under denitrifying conditions (Table 4). The expression of genes involved in heme cofactor biosynthesis required for Nor (Hino et al., 2010) was higher under denitrifying conditions. This indicates that the expression of genes involved in denitrification was upregulated in 6Ca8 cells.

**TABLE 4:** Expression of genes related to aerobic respiration and denirification.

Gene ID	Gene	Definition	TPM value				Fold (denitrification/ aerobic)
			Denitrification condition		Aerobic condition		
			Mean	SD	Mean	SD	
**Assimilately and dissimilately nitrate reduction and denitrification**							
CBBG8_28440	*nirB*	Nitrite reductase large subunit	11.97	4.45	13.66	1.35	0.88
CBBG8_28450	*nirD*	Nitrite reductase [NAD(P)H] small subunit	8.60	1.49	5.37	3.30	1.60
CBBG8_28470	*nasA*	Nitrate reductase	5.20	1.70	6.36	0.75	0.82
CBBG8_49880	*nirV*	Nitrate reductase	445.83	33.08	15.50	5.31	28.76**
CBBG8_49890	*nirK*	Nitrite reductase, copper-containing	1335.34	118.22	52.65	16.42	25.36**
CBBG8_49930	*norD*	Nitric oxide reductase NorD protein	429.39	42.43	16.21	4.35	26.49**
CBBG8_49940	*norQ*	Nitric oxide reductase NorQ protein	889.01	186.78	32.57	7.09	27.29*
CBBG8_49950	*norB*	Nitric-oxide reductase large subunit	1739.85	294.76	43.79	12.46	39.73**
CBBG8_49960	*norC*	Cytochrome *c*	2650.67	307.23	50.08	17.10	52.93**
CBBG8_49970	*norF*	NorF protein	249.76	25.40	8.10	5.44	30.85**
CBBG8_49980	*norE*	Nitric oxide reductase NorE protein	379.16	13.40	13.21	6.30	28.70**
CBBG8_50130	*napE*	Periplasmic nitrate reductase, NapE protein	778.69	58.26	299.19	73.04	2.60**
CBBG8_50140	*napF*	Ferredoxin-type protein NapF	535.07	42.80	145.67	40.18	3.67**
CBBG8_50150	*napD*	Glutamate synthase subunit beta	379.32	16.10	86.06	30.59	4.41**
CBBG8_50160	*napA*	Periplasmic nitrate reductase	610.62	50.45	139.72	36.49	4.37**
CBBG8_50170	*napB*	Periplasmic nitrate reductase, electron transfer subunit	421.51	34.59	102.38	22.84	4.12**
CBBG8_50180	*napC*	Cytochrome *c*-type protein	435.66	31.46	123.18	14.37	3.54**
**Electron transfer**							
**Cytochrome *c* biogenesis (Kranz et al., 2009)**							
CBBG8_06730	*cycH*	*c*-Type cytochrome biogenesis protein CcmI	215.49	10.80	119.07	8.70	1.81**
CBBG8_06740	*ccmE*	Cytochrome *c*-type biogenesis protein CcmE	228.81	20.67	106.16	6.68	2.16**
CBBG8_06750	*cycK*	*c*-Type cytochrome biogenesis protein CcmF	169.57	4.98	94.46	10.00	1.80**
CBBG8_06760	*cycL*	Cytochrome *c*-type biogenesis protein CcmH	103.54	8.29	56.00	9.04	1.85**
CBBG8_23740	*ccmA*	Cytochrome *c* biogenesis ATP-binding export protein CcmA	73.97	7.18	79.63	12.60	0.93
CBBG8_23750	*ccmB*	Heme exporter protein B	30.64	5.74	43.39	5.22	0.71*
CBBG8_23760	*ccmC*	ABC transporter membrane spanning protein (heme)	238.18	31.51	141.69	10.95	1.68**
CBBG8_23770	*ccmD*	Heme exporter protein CcmD	92.56	6.65	41.62	11.77	2.22**
CBBG8_23780	*ccmG*	Thiol:disulfide interchange protein CycY	142.42	11.00	82.88	6.98	1.72**
**Ubiquinone biosynthesis (Kawamukai, 2002)**							
CBBG8_03510	*ubiA*	4-Hydroxybenzoate octaprenyltransferase	81.06	10.62	80.15	2.35	1.01
CBBG8_05030		Demethoxyubiquinone hydroxylase family protein	20.28	5.74	23.82	2.97	0.85
CBBG8_14390	*ubiH*	2-Octaprenyl-6-methoxyphenol hydroxylase	263.22	33.27	83.98	10.52	3.13**
CBBG8_28180	*aarF*	Putative protein kinase UbiB	133.69	7.82	112.08	2.32	1.19*
CBBG8_28190	*ubiE*	Ubiquinone/menaquinone biosynthesis *C*-methyltransferase UbiE	234.60	7.55	162.40	9.39	1.44**
CBBG8_32050	*ubiG*	Ubiquinone biosynthesis *O*-methyltransferase	101.16	5.09	79.89	5.99	1.27**
CBBG8_50010	*ubiX*	Flavin prenyltransferase UbiX	119.91	13.42	4.94	3.61	24.25**
CBBG8_50020	*ubiD*	3-Octaprenyl-4-hydroxybenzoate carboxy-lyase	168.81	12.70	5.44	3.63	31.01**
**Pseudoazurin**							
CBBG8_19460		Pseudoazurin	3029.19	234.41	317.34	86.19	9.55**
**Transcriptional regulator (Baek et al., 2008)**							
CBBG8_12550	*fnrN*	Crp/Fnr family transcriptional regulator	135.16	6.90	87.77	11.81	1.54**
CBBG8_20650	*sinR*	Crp/Fnr family transcriptional regulator	2237.15	133.62	355.52	179.19	6.29**
CBBG8_25710	*actR*	ActR/PrrA/RegA family redox response regulator transcription factor	218.77	11.93	198.12	21.21	1.10
CBBG8_49870	*nnrR*	Crp/Fnr family transcriptional regulator	209.79	12.88	102.91	9.73	2.04**
CBBG8_49900	*nnrS*	NnrS family protein	803.16	114.93	13.20	4.29	60.84**
CBBG8_50000	*nnrU*	Denitrification regulatory protein	410.05	13.49	29.75	3.83	13.79**
**Heme cofactor biosynthesis (Zumft, 1997)**							
CBBG_08500	*hemB*	Delta-aminolevulinic acid dehydratase	487.92	43.99	180.28	13.54	2.71**
CBBG_10990	*cysG*	Uroporphyrinogen-III *C*-methyltransferase	81.81	0.81	115.66	99.63	0.71
CBBG_12540	*hemN*	Coproporphyrinogen-III oxidase	361.10	14.51	74.94	32.03	4.82**
CBBG_19110	*hemF*	Oxygen-dependent coproporphyrinogen-III oxidase	121.67	3.35	127.12	3.10	0.96
CBBG_23000	*hemA*	5-Aminolevulinate synthase	1718.52	32.13	505.02	102.26	3.40**
CBBG_23390	*hemC*	Porphobilinogen deaminase	222.78	9.56	156.89	13.44	1.42**
CBBG_23400	*hemD*	Uroporphyrinogen-III synthase	51.35	6.02	46.13	2.94	1.11
CBBG_25200	*hemJ*	Protoporphyrinogen oxidase HemJ	153.04	6.07	90.63	11.27	1.69**
CBBG_25210	*hemE*	Uroporphyrinogen decarboxylase	327.83	9.56	191.59	11.91	1.71**
CBBG_29570	*hemH*	Ferrochelatase	275.71	27.72	164.23	2.80	1.68*
CBBG_37460	*hemL*	Glutamate-1-semialdehyde 2,1-aminomutase	4.62	1.35	6.18	0.09	0.75
**Oxidative phosphorylation**							
**Cytochrome *c* oxidase**							
CBBG8_04680	*coxB*	Cytochrome *c* oxidase subunit 2	602.62	30.33	446.94	16.27	1.35**
CBBG8_04690	*coxA*	Cytochrome *c* oxidase subunit 1	452.31	23.11	426.00	32.46	1.06
CBBG8_04700	*ctaB*	Protoheme IX farnesyltransferase	198.22	1.96	157.54	19.58	1.26
CBBG8_04730	*coxC*	Cytochrome *c* oxidase subunit 3	472.67	41.34	256.88	9.19	1.84**
CBBG8_26470	*cyoD*	Cytochrome *o* ubiquinol oxidase subunit IV	1043.92	48.51	1001.93	137.40	1.04
CBBG8_26480	*cyoC*	Cytochrome *o* ubiquinol oxidase subunit III	652.19	31.80	628.78	90.79	1.04
CBBG8_26490	*cyoB*	Cytochrome ubiquinol oxidase subunit I	888.71	123.45	868.56	66.65	1.02
CBBG8_26500	*cyoA*	Ubiquinol oxidase subunit 2	1208.30	125.35	982.62	97.81	1.23
**Complex I (NADH dehydrogenase)**							
CBBG8_09470	*nuoA*	NADH-quinone oxidoreductase subunit A	675.63	38.49	991.61	119.08	0.68*
CBBG8_09480	*nuoB*	NADH-quinone oxidoreductase subunit B	1277.58	20.83	1542.76	103.45	0.83*
CBBG8_09490	*nuoC*	NADH-quinone oxidoreductase subunit C	899.34	27.76	1110.21	58.45	0.81**
CBBG8_09510	*nuoD*	NADH-quinone oxidoreductase subunit D	778.15	18.50	1020.79	89.50	0.76*
CBBG8_09530	*nuoE*	NADH dehydrogenase subunit E	843.75	21.23	1150.72	110.18	0.73*
CBBG8_09540	*nuoF*	NADH-quinone oxidoreductase subunit F	839.96	8.79	1073.64	94.80	0.78*
CBBG8_09550	*nuoG*	NADH-quinone oxidoreductase	526.41	33.51	680.45	74.39	0.77*
CBBG8_09560	*nuoH*	NADH-quinone oxidoreductase subunit H	261.06	39.07	381.10	30.49	0.69*
CBBG8_09570	*nuoI*	NADH-quinone oxidoreductase subunit I	433.46	29.18	607.89	68.13	0.71*
CBBG8_09580	*nuoJ*	NADH:ubiquinone oxidoreductase subunit J	512.06	38.89	723.31	48.98	0.71**
CBBG8_09590	*nuoK*	NADH-quinone oxidoreductase subunit K	370.99	26.54	615.33	75.26	0.60**
CBBG8_09600	*nuoL*	NADH:ubiquinone oxidoreductase subunit L	555.31	44.34	827.49	64.48	0.67**
CBBG8_09610	*nuoM*	NADH-quinone oxidoreductase subunit M	492.64	27.91	770.52	63.85	0.64**
CBBG8_09620	*nuoN*	NADH-quinone oxidoreductase subunit N	491.11	20.66	746.94	29.68	0.66**
**Complex III (Cytochrome *bc*_1_ complex)**							
CBBG8_19000	*fbcC*	Cytochrome *c*_1_	624.27	56.29	342.51	21.17	1.82**
CBBG8_19010	*fbcB*	Cytochrome *b*	597.18	69.81	310.39	24.43	1.92**
CBBG8_19020	*fbcF*	Ubiquinol-cytochrome *c* reductase iron-sulfur subunit	583.47	53.20	248.53	6.25	2.35**
**Complex IV**							
**Cytochrome *c* oxidase, *bcc*_3_-type**							
CBBG8_12030	*fixP*	Cytochrome-c oxidase, *cbb*_3_-type subunit III	1104.82	71.57	180.79	73.93	6.11**
CBBG8_12040	*fixQ*	*cbb*_3_-Type cytochrome *c* oxidase subunit IV	302.59	32.81	53.41	25.51	5.67**
CBBG8_12050	*fixO*	Cytochrome c oxidase, *cbb*_3_-type subunit II	828.61	75.55	139.54	61.91	5.94**
CBBG8_12060	*fixN*	Cytochrome c oxidase, *cbb*_3_-type subunit I	1004.64	78.87	168.20	79.70	5.97**
**Cytochrome *bd* complex**							
CBBG8_45120	*cydX*	Cytochrome *bd*_I_ oxidase subunit CydX	65.72	9.44	5.22	4.59	12.58**
CBBG8_45130	*cydB*	Cytochrome *d* ubiquinol oxidase subunit II	562.49	85.74	97.84	31.47	5.75**
CBBG8_45140	*cydA*	Cytochrome *bd* ubiquinol oxidase subunit I	672.13	63.98	127.98	48.74	5.25**
CBBG8_48110	*qxtB*	Ubiquinol oxidase subunit II, cyanide insensitive	319.02	27.45	66.50	8.74	4.80**
CBBG8_48120	*qxtA*	Cytochrome ubiquinol oxidase subunit I	286.91	26.33	60.26	13.64	4.76**

**p < 0.01;

*p < 0.05.

Electron transport in the respiratory chain shifted from aerobic respiration to denitrification (Table 4). Under denitrifying conditions, the expression of genes encoding NADH hydrogenases (Complex I), representative enzymes for aerobic respiration, decreased, while that of genes encoding microaerobic respiratory enzymes of Complex IV, the *cbb_3_-* and *bd-*type cytochrome oxidases (Buschmann et al., 2010; Safarian et al., 2016), increased. Nevertheless, the expression of Complex I genes remained high and was comparable to that of denitrification genes, as indicated by the TPM values (Table 4). These results indicate that denitrification and aerobic respiration occurred simultaneously under conditions mimicking the phyllosphere environment.

**FIGURE 3:**
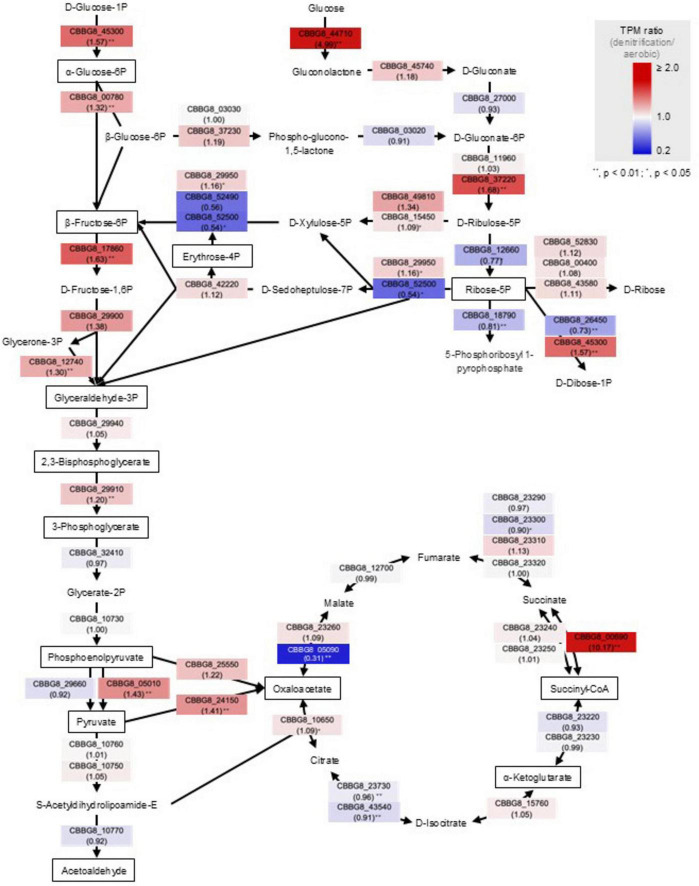
Central metabolic pathways of *Agrobacterium* sp. strain 6Ca8. The ratio of gene expression (based on TPM values) under denitrifying and aerobic conditions is given below the gene ID. A *p*-value of less than 0.01 and 0.05 was indicated as ** and *, respectively. The solid squares indicate the 13 precursor metabolites.

In the central metabolic pathways, namely, the glycolysis and pentose phosphate pathways and the tricarboxylic acid (TCA) cycle, the expression of genes required to transform the primary precursors essential for cellular biosynthesis, including amino acids, nucleotides, and lipids, and for ATP production was altered (Figure 3; Supplementary Table 5). These results suggest that denitrifiers alter gene expression to produce energy and to initiate cellular biosynthesis, by changing the respiration mode to use either oxygen, nitrate, or both.

Simultaneous aerobic and anaerobic respiration has been reported in facultative anaerobic bacteria such as *Escherichia coli* (Basan et al., 2015). Microorganisms select the optimal ATP-producing respiration processes depending on the oxygen concentration to optimize their growth rate while minimizing proteomic costs (Basan et al., 2015). If nitrate can be utilized more easily than oxygen as an electron acceptor, denitrification can occur even in the presence of oxygen. Therefore, the co-expression of the genes for denitrification and aerobic respiration observed here is consistent with previous findings. In agreement with this, Chen and Strous (2013) reported that aerobic respiration and denitrification can occur simultaneously if the oxygen concentration around the bacterial cells is kept very low via oxygen consumption by actively respiring microorganisms, even when the oxygen concentration in the gas phase is relatively high. Parkin (Parkin, 1987) estimated that a water or microbial film at least 160-μm thick could achieve anaerobic conditions at the leaf surface. Such conditions can occur in the phyllosphere of senescent cabbage leaves in the fields: the leaves are rich in organic carbon and nitrate as electron donors and acceptors, respectively, thereby promoting the consumption of ambient oxygen and consequently, inducing denitrification. Using optimal pathways to generate the primary precursors for cellular biosynthesis and ATP production enables microbes to minimize metabolic costs and conserve energy (Noor et al., 2010).

### 3.4 Available carbon for denitrification in the phyllosphere of senescent cabbage leaves

The ecological significance of carbon sources in terms of electron supply for denitrification remains poorly understood. We analyzed carbon sources in the CE medium to identify the electron donors used for denitrification. The late-log phase 6Ca8 culture (at 24 h) (Figure 2A) in CE medium was subjected to NMR analysis. The CE medium contained low-molecular weight compounds, such as amino acids, simple organic acids, and sugars (Supplementary Table 6). Glucose, fructose, and sucrose, and components of the TCA cycle (i.e., malate and succinate) were consumed during incubation. Gluconate, methylguanidine, pyroglutamate, and aspartate were also consumed, while pyruvate, formate, acetate, and mannose were accumulated.

The 15 compounds consumed in the culture were further evaluated for availability. When fructose, glucose, pyroglutamate, succinate, and sucrose were used as sole sources of carbons, cell growth, N_2_O production, and nitrate consumption was observed (Table 5). These five substrates were consumed simultaneously (Supplementary Table 7), indicating that they acted as electron donors for denitrification in the phyllosphere of senescent cabbage leaves. We did not include malate or aspartate as electron donors because nitrate was not significantly consumed (malate, *P* = 0.46; aspartate, *P* = 0.69).

**TABLE 5:** Carbon substrate utilized for denitrification.

Substrate	Cell growth		N_2_O production		Nitrate consumption (mM)	
	O.D._600_		(N_2_O-N μg h^–1^)			
	Mean	SD	Mean	SD	Mean	SD
L-Alanine	0.055	0.004	nd		-1.037	1.307
L-Aspartate	0.151	0.013	0.11	0.18	-0.165	0.738
4-Aminobutyrate	0.038	0	0.26	0.45	0.151	0.555
Citrate^†^	-0.002	0.004	nd		0.151	0.549
Coline^‡^	0.012	0.007	nd		0.085	0.347
Ethanol	0.03	0.013	nd		-0.245	1.165
Ethanolamine	-0.002	0.004	nd		-0.153	0.27
Fructose	0.36	0.011	15.05	8.77	3.751	0.432**
Gluconate^†^	0.031	0.003	nd		0.057	0.259
Glucose	0.308	0.024	16.56	4.15	3.73	0.437**
L-Malate^†^	0.19	0.005	12.09	7.24	0.545	0.441
Methanol	0.006	0.001	0.32	0.55	0.066	0.161
Methylguanidine	0.017	0.003	nd		0.716	2.656
L-Pyroglutamate^†^	0.212	0.017	3.76	0.7	1.857	0.207**
Succinate	0.228	0.003	20.05	5.47	1.946	0.04**
Sucrose	0.363	0.027	17.03	3.84	3.506	0.259**
L-Threonine	0.005	0.006	0.17	0.3	0.867	0.086

**p < 0.01;

*p < 0.05.

† Sodium;

‡ Cloride.

As substrates for denitrification, glucose and succinate are known to have high-energy yields, as reflected in the following formulae:

5C_6_H_12_O_6_ + 24NO_3_^–^ + 24H^+^ → 12N_2_ + 30CO_2_ + 42H_2_O

(Δ*G*°′ = –2,670 kJ mol^–1^) (Strohm et al., 2007)

C_4_H_6_O_4_ + 2.8NO_3_^–^ + 2.8H^+^ → 1.4N_2_ + 4CO_2_ + 4.4H_2_O

(Δ*G*°′ = –1507.59 kJ mol^–1^) (Maier and Pepper, 2015)

Pyroglutamate is widely used as a precursor for the synthesis of other amino acids. Although there are no reports relating pyroglutamate to denitrification, glutamate, a pyroglutamate derivative, is a common substrate for denitrification. The Gibbs free energy of the formation (*Gf*°) of glutamate is comparable to that of succinate (*Gf*°: glucose, -917.22 kJ mol^–1^; succinate, -690.23 kJ mol^–1^; and glutamate, -699.6 kJ mol^–1^) (Madigan et al., 2014). In cabbage crops, the accumulation of succinate, glutamate, and malate is linked to nitrate uptake (Turan and Sevimli, 2005). Thus, cabbage leaves have the potential to become preferred sites for denitrification and N_2_O emission, by accumulating both electron donors and acceptors. We assumed that the other two electron donors, fructose and sucrose, are utilized similarly to glucose, as they are glucose derivatives. This assumption is supported by our RNA-seq analysis showing significant expression of the genes involved in the conversion of sucrose to glucose via fructose (*aglA* and *xylA*) under denitrifying conditions (Supplementary Table 5).

Our study is the first to identify the electron donors enabling denitrification in crops. Although we analyzed low-molecular weight carbon substrates, these can also have high molecular weights. Further studies are needed to reveal other electron donors—the hidden drivers for denitrification.

### 3.5 Denitrifier community in the phyllosphere of senescent cabbage leaves

We analyzed the genomes of the isolated denitrifiers: *Agrobacterium* spp. strains 6Ca8, 5Ca39, and 5Ca50 (Supplementary Figure 6A). In the genome of strains 6Ca8 and 5Ca39, *nap*, *nir*, and *nor* formed a genetic cluster (Supplementary Figure 6B). This cluster has been found in the representative denitrifier, *A. fabrum* C58, and is commonly distributed in other denitrifying strains of *Agrobacterium* spp. and related genera, according to the Agrogenom database (Lassalle et al., 2017).^4^ In contrast, the genome of strain 5Ca50 encoded the N_2_O reductase genes *nosRZDFYLX*, and organization of the denitrification genes differed from that of 6Ca8 and 5Ca39 genomes: in 5Ca50, *nir* and *nor* are encoded on a large chromosome and *nap* and *nos* on a small chromosome. Presence or absence of *nos* in the genome of 5Ca50 and 6Ca8 was supported by culturing experiments, which confirmed that 5Ca50 has N_2_O reductase activity, and that N_2_O is the end product of denitrification by strain 6Ca8.

To elucidate the distributions of 6Ca8, 5Ca39, and 5Ca50 in the phyllosphere of senescent cabbage leaves, we analyzed the bacterial community structure of the senescent leaves and the relative abundances of the these three isolated denitrifiers, based on 16S rRNA gene amplicon sequencing (Supplementary Figure 5 and Table 3). Sixteen bacterial orders were identified as dominant in the cabbage leaf phyllosphere. Alteromonadales, Burkholderiales, and Caedibacterales dominated in the senescent leaves, while Rhodobacterales and Exiguobacterales dominated in the fresh leaves (Supplementary Figure 5). ASVs identical to strains 6Ca8 and 5Ca50 were detected in up to 5.30 and 3.37%, respectively, of the senescent leaves, while were rare (at up to 0.641%) in fresh leaves (Table 3). Considering that the bacterial population increased explosively in the senescent leaves (based on the 16S rRNA gene copies; Table 3), these two strains might develop their populations due to plant senescence. No ASV of strain 5Ca39 was detected in any of the samples, suggesting that this strain is a minor denitrifier.

Accumulating evidence indicates that denitrification is a modular process performed by denitrifiers with partial or complete nitrate- or nitrite-respiration pathways (Lycus et al., 2017). Our genome analysis indicates that 6Ca8 and 5Ca39 produce N_2_O, while 5Ca50 has the potential to reduce N_2_O. Although we did not detect direct evidence of interactions between strains 6Ca8 and 5Ca50, our findings suggest that the phyllosphere of senescent cabbage leaves could be a site for both N_2_O production and reduction, and that their activity and interaction may influence the amount of N_2_O released into the atmosphere.

## 4 Conclusion

In conclusion, we demonstrated that the phyllosphere of senescent leaves directly contributes to N_2_O emissions. N_2_O was emitted as the leaves became senescent and decomposed, and amount of emission varied depending on the position of the leaf (0.10–17.09 μg N_2_O–N h^–1^ g^–1^ fresh weight). Five genera of denitrifiers, such as *Agrobacterium* sp. were present in the phyllosphere, and the representative strain 6Ca8 utilized leaf constituents—specifically nitrate as an electron acceptor and glucose, glucose derivatives, and organic acids as electron donors. The strain simultaneously drove denitrification and aerobic respiration, as indicated by TPM values (249.76–2650.67 for denitrification gene expressions, and 65.72–1277.58 for oxidative phosphorylation gene expressions). Our findings reveal that the phyllosphere of senescent cabbage leaves is a highly reactive microbial system for denitrification that contributes significantly to N_2_O emissions from croplands. This finding implies that not only the soil but also the aboveground leaf residue can be a source of the denitrification process as a microbial function. Our findings make it possible to propose a potential denitrification process as a microbial function in senescing aboveground leaves. Further investigation is needed to clarify the potential of other senescent plants to serve as N_2_O hotspots and the microbial processes involved (e.g., fungal denitrification, nitrification, DNRA, or abiotic processes). At the same time, it is necessary to clarify how denitrifiers shift between respiration modes (aerobic respiration and denitrification) in response to environmental changes within senescent leaves. Such undertaking will help to expand our understanding of the broader picture of the whole niche of microbial nitrogen cycling including the soil and the plant phyllosphere.

## Data Availability

The annotated genomes of strains 6Ca8, 5Ca39, and 5Ca50 have been deposited in the DDBJ nucleotide sequence database under accession numbers AP026433-AP026441. The raw amplicon sequencing data have been deposited in the DRA databases under accession number DRA014294. The RNA-seq nucleotide sequences have been deposited in the DRA databases under accession number DRA014295. The raw NMR spectra have been deposited in the MetaboBank databases under accession number MTBKS225. The remaining data generated or analyzed during the current study are included in this article and Supplementary material.

## Footnotes

1
http://www.fao.org/faostat/en/#data/GA

2
https://github.com/najoshi/sickle

3
http://www.bioinformatics.babraham.ac.uk/projects/fastqc/

4
http://phylariane.univ-lyon1.fr/db/agrogenom/3
