# Supplementary material for: Phyllosphere of senescent crops as a microbial N2O source
Source: Front Microbiol. 2026 Jan 26;16:1650612. doi: 10.3389/fmicb.2025.1650612 (PMC12883736; doi:10.3389/fmicb.2025.1650612)
Supplement: Supplementary file 1 [file Table_1.docx]

Supplementary Material

# Supplementary Figures and Tables

## Supplementary Figures

**B**

**A**


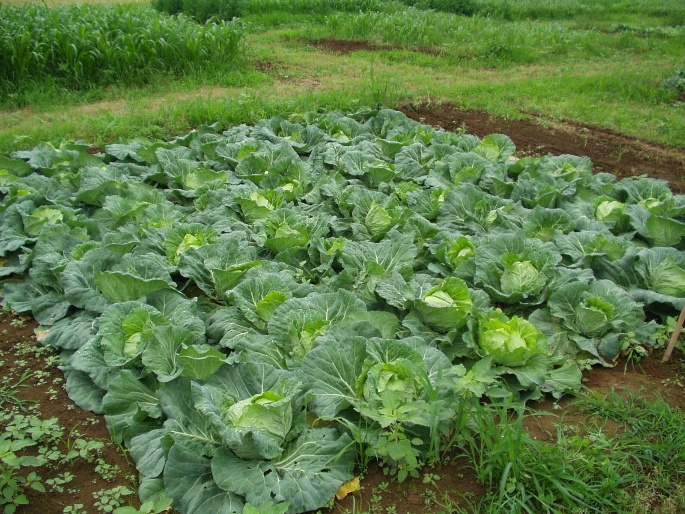

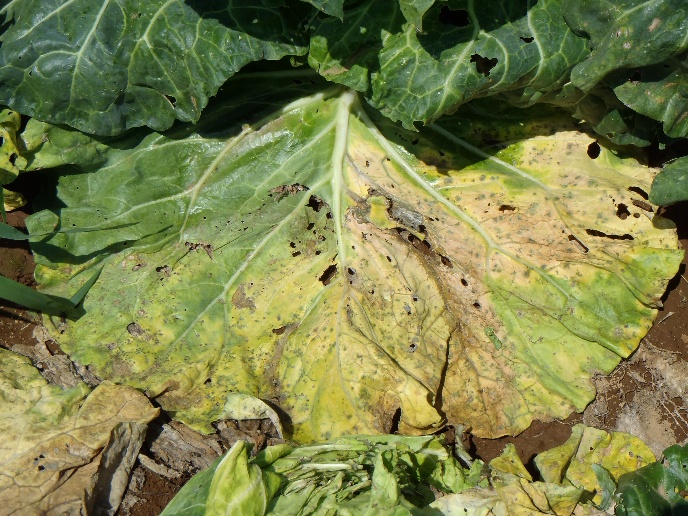


**B**

**A**

**Supplementary Figure 1.** Cabbage field. (A) An experimental plot. (B) Unharvested outer leaves during harvest season.


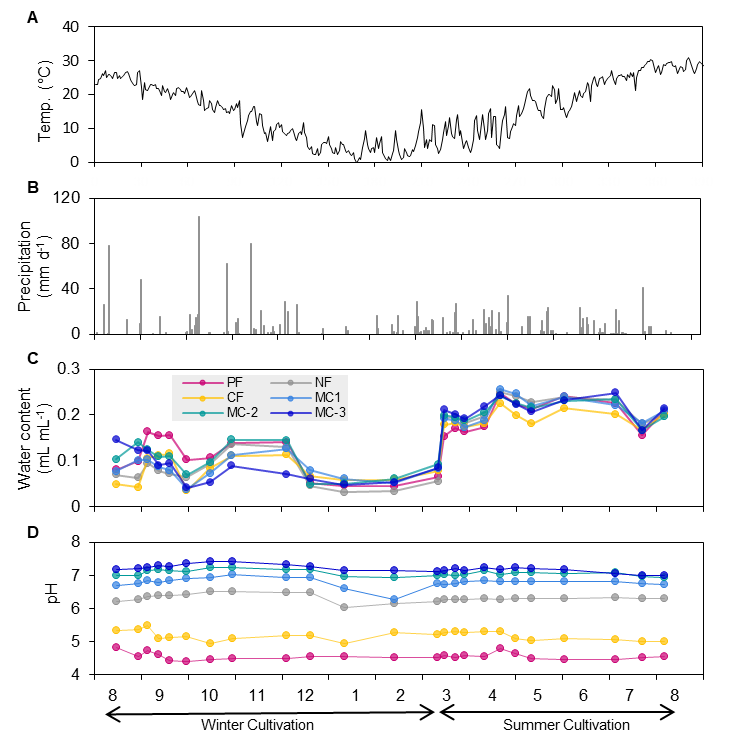


**Supplementary Figure 2.** Environmental factors under long-term treatment with manure, chemical fertilizer, or both, in cabbage field. (A) air temperature; (B) precipitation; (C) soil volumetric water content; (D) soil pH. Symbols: magenta, conventional fertilization with chemical fertilizer and cow manure compost (PF); gray, no fertilizer (NF); yellow, chemical fertilizer (CF); right blue, green, and dark blue, cow manure compost application at 250, 500, and 750 kg ha^−1^, respectively, for total nitrogen (MC-1, -2, and -3).


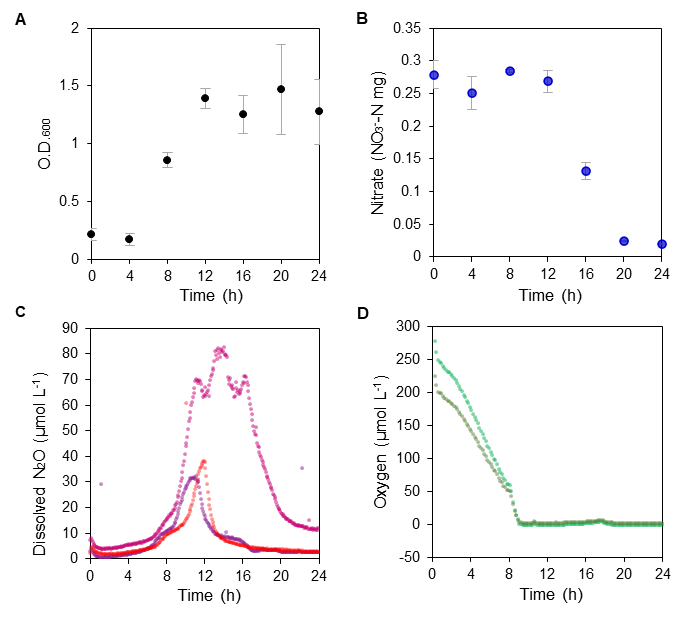


**Supplementary Figure 3.** Growth of the denitrifying *Agrobacterium* sp. strain 6Ca8 on CE medium under aerated conditions. (**A**) Cell growth; (**B**) concentration; (**C**) dissolved N_2_O concentration in the culturenitrate; (**D**) O_2_ concentration.


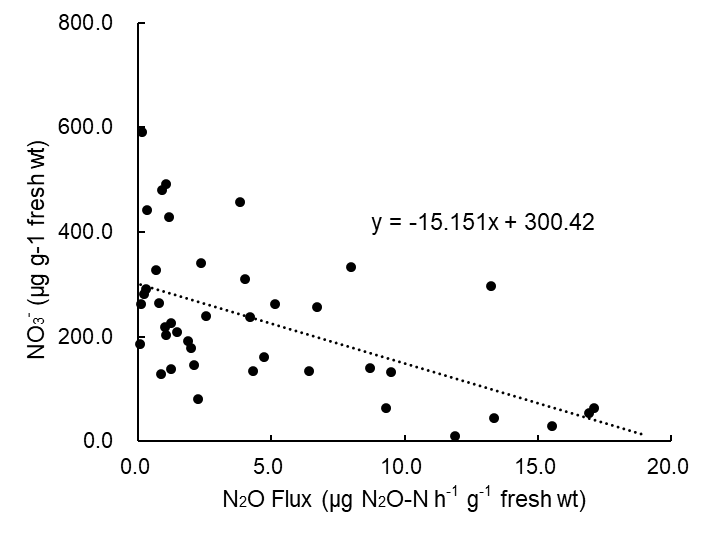


**Supplementary Figure 4.** Relationship between nitrate concentration and N_2_O flux of subsamples of senescent leaf. The values of N_2_O flux (µg N_2_O-N h^-1^ g^-1^ fresh wt) and NO_3_^-^ (µg g^-1^ fresh wt) listed in Table 3 were used for the analysis.


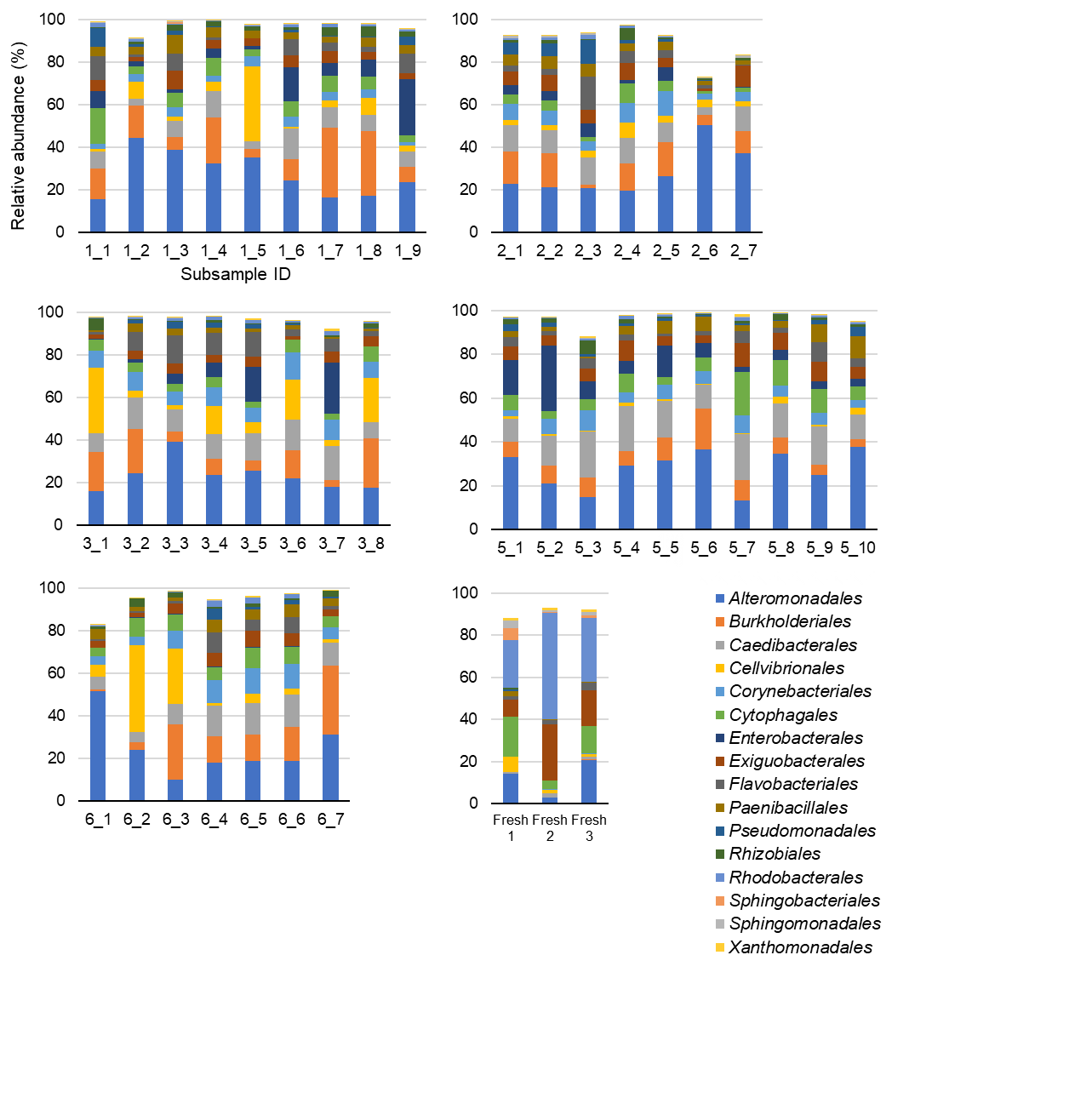


**Supplementary Figure 5.** Bacterial community in the phyllosphere of senescent leaves. The subsample names are same as in Table 3. ASVs in 16 orders, which showed a relative abundance higher than 1% on average in either residual or fresh leaves, were shown. The total abundance of the 16 orders per subsample ranged from 72 .83% (subsample 2_6) to 99.45% (subsample 1_4).

| Strain  **A** | 6Ca8 | 5Ca39 | 5Ca50 | *A. fabrum* C58 |
| --- | --- | --- | --- | --- |
| Total genome size (bp) | 5,671,483 | 5,066,031 | 5,880,176 | 5,674,062 |
| Chromosome (bp) |  |  |  |  |
| Circular | 2,915,820 | 2,824,159 | 3,700,190 | 2,841,490 |
|  | - | - | 985,155 | - |
| Linear | 2,054,514 | 2,241,872 | - | 2,075,560 |
| Megareplicon | - | - | 1,014,566 |  |
| Plasmid (bp) | 701,149 | - | 180,265 | 542,779 (Plasmid pAt) |
|  |  |  |  | 214,233 (Plasmid pTi) |
| G+C content (%) | 58.5 | 59.2 | 55.0 | 58.1 |
| No. of CDSs | 5,378 | 4,816 | 5,519 | 5,419 |
| Coding Ratio (%) | 87.5 | 87.9 | 86.8 | 88.3 |
| No. of rRNA operons | 5 | 4 | 5 | 4 |
| No. of tRNAs | 57 | 57 | 59 | 53 |

**B**

**6Ca8: Plasmid**

**6Ca8: Plasmid**

289157-317734bp

289157-317734bp

***nnrR***

***nnrR***

***nirV***

***nirV***

***norD***

***norD***

***nirK***

***nirK***

***norQ***

***norQ***

***norB***

***norB***

***norC***

***norC***

***norE***

***norE***

***norF***

***norF***

***napA***

***napA***

***napB***

***napB***

***napC***

***napC***

***napD***

***napD***

***napF***

***napF***

***napE***

***napE***

***nnrU***

***nnrU***

**5Ca39: Linear Chromosome**

**5Ca39: Linear Chromosome**

***nnrR***

***nnrR***

***nirV***

***nirV***

***norD***

***norD***

***nirK***

***nirK***

***norQ***

***norQ***

***norB***

***norB***

***norC***

***norC***

***norE***

***norE***

***norF***

***norF***

***napA***

***napA***

***napB***

***napB***

***napC***

***napC***

***napD***

***napD***

***napE***

***napE***

***napF***

***napF***

***nnrU***

***nnrU***

418444-444892bp

418444-444892bp

***nnrR***

***nnrR***

***nirV***

***nirV***

***nirK***

***nirK***

***norD***

***norD***

***norQ***

***norQ***

***norB***

***norB***

***norC***

***norC***

***norE***

***norE***

***norF***

***norF***

***nnrU***

***nnrU***

**5Ca50: Large chromosome (left) and small chromosome (right)**

**5Ca50: Large chromosome (left) and small chromosome (right)**

***napA***

***napA***

***napB***

***napB***

***napC***

***napC***

***napD***

***napD***

***napE***

***napE***

***nosR***

***nosR***

***nosZ***

***nosZ***

***nosD***

***nosD***

***nosF***

***nosF***

***nosY***

***nosY***

***nosL***

***nosL***

***nosX***

***nosX***

2880928-2893823bp

2880928-2893823bp

61879-77413bp

61879-77413bp

***nnrR***

***nnrR***

***nirV***

***nirV***

***norD***

***norD***

***nirK***

***nirK***

***norQ***

***norQ***

***norB***

***norB***

***norC***

***norC***

***norE***

***norE***

***norF***

***norF***

***napA***

***napA***

***napB***

***napB***

***napC***

***napC***

***napD***

***napD***

***napF***

***napF***

***napE***

***napE***

***nnrU***

***nnrU***

**C58: Linear chromosome**

**C58: Linear chromosome**

1517414-1545995bp

1517414-1545995bp

**Supplementary Figure 6.** Genomic features of the isolated denitrifiers. (**A**) Table depicting genomic features of the isolated denitrifiers belonging to *Agrobacterium* sp. (**B**) Organization of the denitrification-related gene clusters in the isolated denitrifier genomes. Arrow represents the direction of gene transcription. The genomic features of a representative denitrifier, *A. fabrum* C58 (Goodner et al., 2001; Wood et al., 2001), are also shown.

## Supplementary Tables

| Treatment | pH | TC (g kg^-1^) | TN (g kg^-1^) | CEC (cmol kg^-1^) | Available N (mg kg^-1^) | Bulk density | Ex. Cation (cmol kg^-1^) | | | Nitrogen application (kgN ha^-1^) | | Cumulative N_2_O emission (mg N_2_O-N m^2 -1^) | Nitrate content in the summer harvest season  (NO_3_^-^-N mg g^-1^ dry weight) | |
| --- | --- | --- | --- | --- | --- | --- | --- | --- | --- | --- | --- | --- | --- | --- |
|  |  |  |  |  |  |  | Ca | Mg | K | Chemical fertilizer^*^ | Cow manure compost^†^ |  | Outer leaf | Soil |
| PF^‡^ | 5.15 | 48.2 | 4.91 | 29.6 | 127 | 0.84 | 0.57 | 0.09 | 0.15 | 250 | 162 | 0.406 | 11.3 | 0.072 |
| NF | 6.38 | 33.8 | 3.15 | 26.2 | 63 | 0.85 | 0.61 | 0.17 | 0.03 | 0 | 0 | 0.025 | 3.4 | 0.001 |
| CF | 4.63 | 36.2 | 3.42 | 24.6 | 71 | 0.85 | 0.24 | 0.03 | 0.07 | 250 | 0 | 0.128 | 10.0 | 0.042 |
| MC-1 | 6.86 | 49 | 4.95 | 32.7 | 146 | 0.83 | 0.94 | 0.31 | 0.17 | 0 | 250 | 0.036 | 3.0 | 0.012 |
| MC-2 | 7.06 | 58.5 | 6.32 | 35.7 | 181 | 0.82 | 1.22 | 0.4 | 0.24 | 0 | 500 | 0.083 | 6.0 | 0.014 |
| MC-3 | 7.24 | 74 | 8.29 | 41.9 | 250 | 0.82 | 1.31 | 0.49 | 0.32 | 0 | 750 | 0.129 | 7.6 | 0.045 |

**Supplementary Table 1.** Chemical characteristics of soil, amount of nitrogen applications, cumulative N_2_O emission, and nitrate content of outer leaf and soil under the summer cultivation harvest season, in the six treatments.

^*^ Chemical fertilizer contained 40 g kg^−1^ of ammonium-nitrogen, 40 g kg^−1^ of nitrate-nitrogen, 17 g kg^−1^ of phosphorous, and 33 g kg^−1^ of potassium.

^†^ Cow manure compost, made from cow excreta and sawdust, contained 70% of water, 27 g kg^−1^ of total nitrogen, and 428 g kg^−1^ of total carbon. The pH (H_2_O) value of the compost was 9.5 (compost:water = 1:10, fresh weight basis).

^‡^ PF was the standard fertilizer treatment recommended by prefectural governments in Japan. The application rates of PF were 250 kg ha^−1^ for nitrogen, 109 kg ha^−1^ for phosphorous, 207 kg ha^−1^ for potassium, and 20 Mg (fresh weight) ha^−1^ for a cow manure compost.

**Supplementary Table 2.** Expression of genes related to denitrification at early and mid-log phases.

|  |  |  | TPM value | | | | | | | | | | | | | |
| --- | --- | --- | --- | --- | --- | --- | --- | --- | --- | --- | --- | --- | --- | --- | --- | --- |
|  |  |  | Early-log phase^†^ | | | | | |  | Mid-log phase^‡^ | | | | | | |
|  |  | Conditions | Denitrification | | Aerobic | | Fold (denitrificat-ion/ aerobic) | |  | Denitrification | | Aerobic | | Fold (denitrificat-ion/ aerobic) | | |
| Gene ID | Gene | Definition | Mean | sd | Mean | sd |  |  |  | Mean | sd | Mean | sd |  |  |  |
| CBBG8_28440 | *nirB* | Nitrite reductase large subunit | 11.97 | 4.45 | 13.66 | 1.35 | 0.88 |  |  | 44.71 | 10.73 | 18.05 | 11.55 | 2.48 | * |  |
| CBBG8_28450 | *nirD* | Nitrite reductase (NAD(P)H) small subunit | 8.60 | 1.49 | 5.37 | 3.30 | 1.60 |  |  | 47.00 | 11.21 | 14.47 | 12.57 | 3.25 | * |  |
| CBBG8_28470 | *nasA* | Nitrate reductase | 5.20 | 1.70 | 6.36 | 0.75 | 0.82 |  |  | 33.81 | 13.26 | 10.07 | 3.54 | 3.36 | * |  |
| CBBG8_49880 | *nirV* | Nitrate reductase | 445.83 | 33.08 | 15.50 | 5.31 | 28.76 | ^**^ |  | 1094.68 | 46.99 | 123.17 | 30.63 | 8.89 | ** |  |
| CBBG8_49890 | *nirK* | Nitrite reductase, copper-containing | 1335.34 | 118.22 | 52.65 | 16.42 | 25.36 | ^**^ |  | 3956.96 | 81.23 | 519.93 | 77.74 | 7.61 | ** |  |
| CBBG8_49930 | *norD* | Nitric oxide reductase NorD protein | 429.39 | 42.43 | 16.21 | 4.35 | 26.49 | ^**^ |  | 474.93 | 24.14 | 168.27 | 17.09 | 2.82 | ** |  |
| CBBG8_49940 | *norQ* | Nitric oxide reductase NorQ protein | 889.01 | 186.78 | 32.57 | 7.09 | 27.29 | ^*^ |  | 948.65 | 48.95 | 333.93 | 58.59 | 2.84 | ** |  |
| CBBG8_49950 | *norB* | Nitric-oxide reductase large subunit | 1739.85 | 294.76 | 43.79 | 12.46 | 39.73 | ^**^ |  | 1638.60 | 35.69 | 471.48 | 98.13 | 3.48 | ** |  |
| CBBG8_49960 | *norC* | Cytochrome *c* | 2650.67 | 307.23 | 50.08 | 17.10 | 52.93 | ^**^ |  | 2181.70 | 96.06 | 490.52 | 92.33 | 4.45 | ** |  |
| CBBG8_49970 | *norF* | NorF protein | 249.76 | 25.40 | 8.10 | 5.44 | 30.85 | ^**^ |  | 254.00 | 38.79 | 53.59 | 5.29 | 4.74 | * |  |
| CBBG8_49980 | *norE* | Nitric oxide reductase NorE protein | 379.16 | 13.40 | 13.21 | 6.30 | 28.70 | ^**^ |  | 431.84 | 12.39 | 88.35 | 3.66 | 4.89 | ** |  |
| CBBG8_50130 | *napE* | Periplasmic nitrate reductase, NapE protein | 778.69 | 58.26 | 299.19 | 73.04 | 2.60 | ^**^ |  | 728.92 | 118.28 | 687.29 | 28.31 | 1.06 |  |  |
| CBBG8_50140 | *napF* | Ferredoxin-type protein NapF | 535.07 | 42.80 | 145.67 | 40.18 | 3.67 | ^**^ |  | 586.60 | 14.86 | 460.18 | 71.68 | 1.27 |  |  |
| CBBG8_50150 | *napD* | Glutamate synthase subunit beta | 379.32 | 16.10 | 86.06 | 30.59 | 4.41 | ^**^ |  | 382.65 | 44.82 | 273.26 | 13.09 | 1.40 | * |  |
| CBBG8_50160 | *napA* | Periplasmic nitrate reductase | 610.62 | 50.45 | 139.72 | 36.49 | 4.37 | ^**^ |  | 696.43 | 15.12 | 512.22 | 21.06 | 1.36 | ** |  |
| CBBG8_50170 | *napB* | Periplasmic nitrate reductase, electron transfer subunit | 421.51 | 34.59 | 102.38 | 22.84 | 4.12 | ^**^ |  | 519.79 | 19.68 | 418.01 | 15.82 | 1.24 | ** |  |
| CBBG8_50180 | *napC* | Cytochrome *c*-type protein | 435.66 | 31.46 | 123.18 | 14.37 | 3.54 | ^**^ |  | 449.02 | 20.62 | 431.00 | 29.96 | 1.04 |  |  |

^**^ p < 0.01, ^*^ p < 0.05.

^†^ O.D._600_＝0.4 under denitrification condition

^‡^ O.D._600_＝0.9 under denitrification condition

| Nearest species and accession number | |  | Number of isolated denitrifiers | | | | | |
| --- | --- | --- | --- | --- | --- | --- | --- | --- |
|  |  |  | CE medium | | | R2A-N medium | | |
|  |  |  | Subsample ID^*^ | | | Subsample ID | | |
|  |  | % Similarity | 1_7 | 5_7 | 6_3 | 1_7 | 5_7 | 6_3 |
| *Achromobacter denitrificans* strain DSM 30026 | NR_042021 | 99 |  |  | 1 |  |  |  |
| *Agrobacterium fabacearum* strain CNPSo 675 | NR_174322 | 99 |  | 1 |  | 1 | 2 |  |
| *Agrobacterium tumefaciens* strain NCPPB2437 | NR_115516 | 100 |  | 1 |  |  |  |  |
| *Agrobacterium fabacearum* strain CNPSo 675 | NR_174322 | 99 |  |  | 9 |  |  |  |
| *Alcaligenes faecalis* strain NBRC 13111 | NR_113606 | 99 |  |  |  | 21 |  | 1 |
| *Alcaligenes aquatilis* strain LMG 22996 | NR_114959 | 99 |  |  |  |  | 1 |  |
| *Brucella anthropi* strain ATCC 49188 | NR_074243 | 99 | 1 |  |  |  |  |  |
| *Stenotrophomonas maltophilia* strain IAM 12423 | NR_041577 | 99 | 2 | 2 |  | 3 |  |  |

**Supplementary Table 3.** Classification of denitrifiers isolated from senescent cabbage phyllosphere by 16S rRNA gene sequencing analysis.

^*^ The denitrifiers were isolated from the senescent leaf subsamples, 1_7, 5_7, and 6_3, which showed high N_2_O emissions (Table 1).

**Supplementary Table 4.** Top 20 mRNA sequences of strain 6Ca8 that were highly expressed under denitrification conditions.

|  |  |  | TPM value | | | | Fold (denitrificati-on/ aerobic) |  |
| --- | --- | --- | --- | --- | --- | --- | --- | --- |
|  |  |  | Denitrification condition | | Aerobic condition | |  |  |
| Gene ID | Gene | Definition | Mean | sd | Mean | sd |  |  |
| CBBG8_49900 |  | NnrS family protein | 803.16 | 114.93 | 13.20 | 4.29 | 60.84 | ^**^ |
| CBBG8_49960 | *norC* | Cytochrome *c* | 2650.67 | 307.23 | 50.08 | 17.10 | 52.93 | ^**^ |
| CBBG8_50070 |  | Hypothetical protein | 189.77 | 6.48 | 4.24 | 3.67 | 44.76 | ^**^ |
| CBBG8_49950 | *norB* | Nitric-oxide reductase large subunit | 1739.85 | 294.76 | 43.79 | 12.46 | 39.73 | ^**^ |
| CBBG8_50080 |  | TonB-dependent receptor | 718.71 | 93.47 | 21.80 | 6.07 | 32.97 | ^**^ |
| CBBG8_50040 |  | SCP2 domain-containing protein | 308.03 | 11.74 | 9.42 | 4.88 | 32.71 | ^**^ |
| CBBG8_50020 | *ubiD* | 3-Octaprenyl-4-hydroxybenzoate carboxy-lyase | 168.81 | 12.70 | 5.44 | 3.63 | 31.01 | ^**^ |
| CBBG8_49970 | *norF* | NorF protein | 249.76 | 25.40 | 8.10 | 5.44 | 30.85 | ^**^ |
| CBBG8_49990 |  | Hypothetical protein | 480.99 | 48.02 | 15.72 | 8.49 | 30.60 | ^**^ |
| CBBG8_49880 | *nirV* | Nitrate reductase | 445.83 | 33.08 | 15.50 | 5.31 | 28.76 | ^**^ |
| CBBG8_49980 | *norE* | Nitric oxide reductase NorE protein | 379.16 | 13.40 | 13.21 | 6.30 | 28.70 | ^**^ |
| CBBG8_50060 |  | U32 Family peptidase | 210.20 | 5.25 | 7.51 | 4.13 | 27.99 | ^**^ |
| CBBG8_49940 | *norQ* | Nitric oxide reductase NorQ protein | 889.01 | 186.78 | 32.57 | 7.09 | 27.29 | ^**^ |
| CBBG8_49930 | *norD* | Nitric oxide reductase NorD protein | 429.39 | 42.43 | 16.21 | 4.35 | 26.49 | ^**^ |
| CBBG8_49890 | *nirK* | Nitrite reductase, copper-containing | 1335.34 | 118.22 | 52.65 | 16.42 | 25.36 | ^**^ |
| CBBG8_49920 |  | DUF2249 domain-containing protein | 1549.71 | 100.62 | 61.26 | 14.06 | 25.30 | ^**^ |
| CBBG8_50010 | *ubiX* | Flavin prenyltransferase UbiX | 119.91 | 13.42 | 4.94 | 3.61 | 24.25 | ^**^ |
| CBBG8_50090 |  | Nitrate ABC transporter substrate-binding protein | 517.80 | 65.08 | 22.05 | 6.20 | 23.49 | ^**^ |
| CBBG8_53300 | *hspC* | Molecular chaperone Hsp20 | 1022.31 | 65.18 | 53.07 | 16.25 | 19.26 | ^**^ |
| CBBG8_50100 |  | ABC transporter permease | 327.89 | 62.84 | 17.59 | 3.20 | 18.64 | ^**^ |

^**^, p < 0.01; ^*^, p < 0.05.

|  | Concentration (mM) | | | |  |
| --- | --- | --- | --- | --- | --- |
|  | 0h | | 20h | |  |
|  | Mean | sd | Mean | sd |  |
| Glucose | 4.57 | 0.09 | 1.46 | 0.16 | ^**^ |
| Fructose | 5.16 | 0.15 | 1.44 | 0.15 | ^**^ |
| Sucrose | 4.90 | 0.14 | 2.24 | 0.22 | ^**^ |
| Pyroglutamate | 4.32 | 0.12 | 1.61 | 0.35 | ^**^ |
| Succinate | 3.90 | 0.06 | -0.09 | 0.02 | ^**^ |

**Supplementary Table 7.** Consumption of electron donor.

^**^ p < 0.01, ^*^ p < 0.05.

**References**

Goodner, B., Hinkle, G., Gattung, S., Miller, N., Blanchard, M., Qurollo, B., et al. (2001). Genome sequence of the plant pathogen and biotechnology agent *Agrobacterium tumefaciens* C58. *Science,* 294**,** 2323-2328. https://:doi.org/10.1126/science.1066803.

Wood, D. W., Setubal, J. C., Kaul, R., Monks, D. E., Kitajima, J. P., Okura, V. K., et al. (2001). The genome of the natural genetic engineer *Agrobacterium tumefaciens* C58. *Science,* 294**,** 2317-2323. https://:doi.org/10.1126/science.1066804.
